# Medical Infrared Thermography in Odontogenic Facial Cellulitis as a Clinical Decision Support Tool. A Technical Note

**DOI:** 10.3390/diagnostics11112045

**Published:** 2021-11-04

**Authors:** Stéphane Derruau, Fabien Bogard, Guillaume Exartier-Menard, Cédric Mauprivez, Guillaume Polidori

**Affiliations:** 1 UFR Odontologie, Université de Reims Champagne-Ardenne, 51100 Reims, France; guillaume.exartier-menard@univ-reims.fr (G.E.-M.); cedric.mauprivez@univ-reims.fr (C.M.); 2 Pôle de Médecine Bucco-Dentaire, Service de Chirurgie Orale, Centre Hospitalier Universitaire de Reims, 51092 Reims, France; 3 BioSpecT EA-7506, UFR Pharmacie, Université de Reims Champagne-Ardenne, 51096 Reims, France; 4 MATIM EA, UFR Sciences, Université de Reims Champagne-Ardenne, 51687 Reims, France; fabien.bogard@univ-reims.fr (F.B.); guillaume.polidori@univ-reims.fr (G.P.); 5 BIOS EA-4691, UFR Pharmacie, Université de Reims Champagne-Ardenne, 51096 Reims, France

**Keywords:** medical infrared thermography, odontogenic cellulitis, head and neck infection, clinical decision support tool

## Abstract

Background: Odontogenic cellulitis are frequent infections of the head and neck fascial spaces that can sometimes spread and be life-threatening, requiring urgent hospitalization. Early diagnosis of facial cellulitis with diffuse inflammatory process is crucial in patient management but not always obvious in the field. Medical infrared thermography (MIT) is a noninvasive tool increasingly used to evaluate skin temperature maps and delineate inflammatory lesions. Objective: The aim of this work was to evaluate the use of MIT to improve the clinical examination of patients with facial cellulitis. Methods: Image processing work was carried out to highlight the thermal gradient resulting from inflammation linked to infection, in 2 patients with facial cellulitis. Results: In real-time, MIT allowed to precisely locate the inflammatory focus linked to cellulitis with no propagation to danger areas such as infraorbital space or around pharyngeal axis. Conclusions: Here, we show the first cases using MIT as a powerful complementary tool in the clinical evaluation of patients with facial cellulitis. Significance: This technology could help optimize the hospitalization decision through a facilitated assessment of infection spread in head and neck tissues and helping to incision for drainage.

## 1. Introduction

Tooth infections are a common presenting complaint in dental offices and emergency departments (ED). These minor localized pyogenic infections can progress to the fascial spaces of the head and neck when neglected, leading to the establishment of cellulitis [1]. Facial cellulitis is noted in prior studies to represent 2.7% to 3.4% of all dental ED visits in hospital-based settings [1,2,3]. Although 94.5% of dental ED patients are routinely discharged, these emergencies lead to many hospitalizations each year in the United States [1], including approximately 37,000 for facial cellulitis [4]. Despite IV antibiotic therapy and surgical management, odontogenic infections may promptly spread to deep spaces of the head and neck. Serious complications can then arise and be life-threatening, such as critical airway obstructions, downward mediastinitis, cavernous sinus thrombosis or septic shock [5,6,7,8]. Inpatient mortality was estimated to be between 0.01% to 0.2%, associated with advanced age of the patient, the presence of comorbid factors such as diabetes, and delayed surgical treatment [1,4,8]. Therefore, a critical step in management of these potentially fatal infections is the recognition of the source of infection, the various clinical presentations of the infection (abscess, cellulitis, and necrotizing fasciitis), and the accuracy with which the number of fascial spaces involved is determined [4,7,8,9,10]. Early extraction of the offending tooth and drainage during the acute phase of facial cellulitis have been shown that reduce the need for more invasive surgical procedures and possible hospitalization, which are major drivers of healthcare costs [4,5,11]. However, the clinical examination of a swollen face to differentiate localized/diffuse cellulitis from an abscess and to identify the affected anatomical spaces may be difficult [12]. Indeed, palpation of the skin is often painful, and is sometimes not possible, e.g., with children. In addition, the extent of the infection can be misjudged, with fluctuations of purulent accumulation sometimes being masked or even absent, especially following the administration of nonsteroidal anti-inflammatory drugs or empiric antibiotic therapy without associated dental intervention, or in obese patients [8,13]. Previous studies have found that clinical examination was able to identify a drainable collection in 33% to 76% of cases [7,9]. Ultrasonography (USG) [14] or computed tomography (CT) scan [8,12] are therefore useful in identifying purulent accumulation. However, these medical imaging techniques are not always available at the first presentation of the patient in the emergency ward. Swelling is a main clinical feature of facial cellulitis, often accompanied by other classic signs of inflammation, i.e., tenderness, redness and warmth. 

Infrared thermography is a nonradiative, noncontact, noninvasive and fast imaging technique that allows passive observation of thermal radiation emitted by any object with temperature above absolute zero. Infrared thermography provides functional anatomical information, because temperature is a useful indicator of disease [15,16]. Thermography as a complementary diagnostic support has been applied in medicine since the mid-1990s to detect various inflammatory diseases, complex regional pain syndrome or even Raynaud’s phenomenon [16,17]. This tool also seems to be suitable for diagnoses of infectious processes, e.g., in postoperative wounds after cesarean section, in the feet of diabetics, or in a central venous catheter in children [18,19,20]. In the oral and maxillofacial area, studies have shown its potential for the diagnosis of various diseases such as traumatic fractures, osteomyelitis [21], malignant neoplasms, cervical lymph node metastasis from oral cancer [15], quantification of postsurgical inflammation after removal of wisdom teeth [22] and retained teeth [23], or arthralgia of temporomandibular joints [24]. Surprisingly, thermography has almost never been used for dental cellulitis, which is an inflammatory disease.

To our knowledge, no previous study of thermal imaging has determined whether assessing skin surface temperature could aid in the management of facial cellulitis. The aim of this work is therefore to highlight the potential of MIT as a complementary support tool, both in decisions to hospitalize and regarding the potential efficacy of incisions for drainage.

## 2. Materials and Methods

The methodology adopted to qualify the value of MIT as a complementary aid to the decision to hospitalize a patient with facial cellulitis is an a posteriori data confrontation methodology. In this study, first, clinicians made diagnoses of two clinical cases and proposed the therapeutic treatment that seemed most appropriate. They decided whether or not to hospitalize the patients. At the same time (but independently), thermal scientists produced infrared thermography images and developed an original differential approach allowing the precise localization of the inflamed skin areas. After all treatments were completed and patients had been cured, a comparison was made between all the medical diagnostic tools. The aim was to see if the MIT could be considered as a complementary decision-making tool that could better inform hospitalization decisions in cases where this was questionable.

### 2.1. Participant

Two patients with cervicofacial cellulitis treated in the Oral Medicine Department at the Reims University Hospital (France) were included in this study. The inclusion criteria were that the patients must have been treated for an oral emergency, be over 18 years old, and have health insurance. Exclusion criteria were also defined, e.g., not being an adult protected by law, not being pregnant or breastfeeding. Other exclusion criteria were defined to avoid internal and external metabolic effects unrelated to cellulite which could interfere with the thermal cervico-facial examination. Additionally, patients should not suffer from a depressive disorder, an inflammatory disease known to affect the orofacial sphere, another active inflammatory oral pathology, dysfunction of the masticatory system and should not be undergoing antibiotic treatment. Relevant data on the individual participants was obtained [25]. In addition, no technical factor should affect skin exposure such as the presence of a beard for the realization of thermal images, and the patient had to be able to remain in a static position. These criteria were in accordance with the guidelines for oral systemic thermography from the American Academy of Thermology [26]. 

### 2.2. Thermal Imaging Recording 

Facial skin temperatures were recorded in orthostatic position using a VarioCAM^®^ HD thermal imaging camera with 1024 × 768 pixel resolution (Jenoptik, Altenstadt, Germany) with respect to the standard protocol for infrared imaging in medicine [27,28]. An emissivity factor of 0.98 for the human skin was used to obtain appropriate skin thermographs. The different parts of the optical detector comprise microscopically small thin-film resistors, arranged a few micrometers above the silicon reading circuit. The optical system of the camera is thus able to measure the thermal radiation of the observed scene, thanks to these elements of the detector, which absorb this radiation. The resulting variation in temperature of the detector elements then gives signals that can be analyzed electronically. Analyses of the thermal images were made possible with the use of postprocessing software (IRBIS^®^ 3.1, InfraTec, Dresden, Germany). Each pixel of the image, weighted by its thermal size, was considered, making it possible to obtain matrices which were thermally characteristic of predetermined zones (affected or healthy). 

### 2.3. Thermal Data Processing

A medical thermal image provides a qualitative mapping of the skin temperature field 〈H〉 of a defined region of interest (ROI). In the case of inflamed skin areas, more than reading the local temperature values 〈D〉, it is the thermal level of the inflammation that is necessary to qualify the degree of severity of the disease. This thermal level or thermal gradient can only be obtained if one has prior knowledge of the thermal mapping of the same healthy area. Under normal and healthy conditions, there is thermal symmetry between contralateral regions of the face [29].

Then, the thermal gradient qualifying the severity of inflammation in lateral dental cellulitis may be obtained by subtracting the thermal matrices of the diseased and healthy areas of interest in each hemiface. It should be noted that in a healthy subject, the skin temperatures of the face are not uniform, deriving primarily from the pattern of superficial blood vessels, and also due to the variable nature of the subcutaneous tissues. 

For simplicity, rectangular areas of interest were considered, leading to 2D rectangular matrices, where each element represented the temperature assigned to the corresponding pixel. Consider the following matrices of size (*m*,*n*) of ROI:

(1) $$\mathrm{Healthy}\;\mathrm{temperature}\;\mathrm{matrix}:\;\langle H\rangle ={\left(h_{i,j}\right)}_{\left(i,j\right)\in \left[1,m\right]\times \left[1,n\right]}=\left[\begin{array}{ccc} h_{11} & \cdots & h_{1n}\\ \vdots & \ddots & \vdots \\ h_{m1} & \cdots & h_{mn} \end{array}\right]$$

(2) $$\mathrm{Disease}\;\mathrm{temperature}\;\mathrm{matrix}:\;\langle D\rangle ={\left(d_{i,j}\right)}_{\left(i,j\right)\in \left[1,m\right]\times \left[1,n\right]}=\left[\begin{array}{ccc} d_{11} & \cdots & d_{1n}\\ \vdots & \ddots & \vdots \\ d_{m1} & \cdots & d_{mn} \end{array}\right]$$

To compare pixel by pixel right and left hemifaces, a mirror view of the healthy thermal matrix should be performed:

(3) $$\begin{array}{c} \mathrm{Healthy}\;\mathrm{temperature}\;\mathrm{matrix}\;\mathrm{Equation}\;(1):\;\langle H\rangle ={\left(h_{i,j}\right)}_{\left(i,j\right)}=\left[\begin{array}{ccc} h_{11} & \cdots & h_{1n}\\ \vdots & \ddots & \vdots \\ h_{m1} & \cdots & h_{mn} \end{array}\right]\\ ⇓\\ \mathrm{Mirror}\;\mathrm{healthy}\;\mathrm{temperature}\;\mathrm{matrix}:\;\langle H^{*}\rangle =\left(h_{i,n+1-j}\right)=\left[\begin{array}{ccc} h_{1n} & \cdots & h_{11}\\ \vdots & \ddots & \vdots \\ h_{mn} & \cdots & h_{m1} \end{array}\right] \end{array}$$

Then, it becomes possible to extract, by subtraction, the real disease level:

(4) $$\langle R\rangle =\langle D\rangle -\langle H^{*}\rangle$$

Based on the principle that the site of inflammation is the place where the temperature of the skin is the highest, a specific threshold, $\Delta T_{\mathrm{threshold}}$, was defined for each patient and the severity degree of pathology. The coefficients of the resulting matrix were then assigned the condition:

(5) $$\langle R_{ij}\rangle \;=\;0\;\mathrm{for}\;\langle R_{ij}\rangle <\Delta T_{\mathrm{threshold}}$$

This criterion enabled us to create an accurate localized representation of the disease.

## 3. Results

### 3.1. Case Reports

The two patients with cervicofacial cellulitis included in this study were both women, aged 25 and 32 years, respectively. Clinical information about the patients is summarized in Table 1.

*Patient 1*

A 25-year-old female came overnight to the hospital emergency department for left facial painful swelling involving the left cheek and lower eyelid. The pain was severe and not relieved by acetaminophen and ibuprofen. Dental extraction of left upper and lower third molars had been done three days prior. No antibiotic had been prescribed for the surgical procedure. 

**Figure 1:**
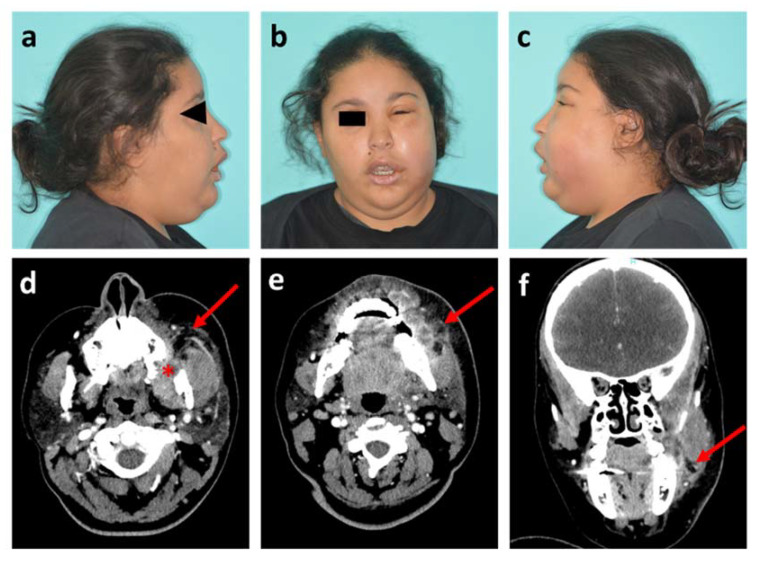
Photographs and head and neck CT scan of patient 1. (**a**–**c**) Visible images of healthy right profile, front view and affected left profile, respectively; (**d**,**e**) Axial plane of CT scan in soft tissue window, at maxillary level with the socket of the tooth #28 (red star) and at mandibular level, respectively; (**f**) Sagittal plane of CT scan. Red arrows show the infectious collection.

A physical examination showed a fever at 38.7 °C and swelling with ill-defined borders of the left buccal space with spread to the lower eyelid. This patient, with a body mass index (BMI) of 28 kg/m^2^, presented a visual disturbance, with a swollen eyelid and absence of proptosis (Figure 1a–c). No obvious sign of fluctuation, crepitus or tenderness (cord-like structure) in the nasolabial fold was detected on palpation. Mouth opening was restricted to 15 mm. No Dysphagia or dyspnea was noted. An intra-oral examination revealed vestibular swelling with a complete obliteration maxillary buccal vestibule. 

Clinical findings showed left maxillary vestibular buccal swelling with infection extending into the buccal and infra-orbital spaces. The white blood cell (WBC) count was 14 × 10^3^/µL and the CRP was 11 mg/L. The patient was hospitalized for the administration of intravenous antibiotics (amoxicillin/clavulanic acid with metronidazole) and a computed tomography (CT) scan of head and neck was performed the next day. This radiological exam confirmed the source of infection as the starting point of the maxillary dental extraction (tooth #28) with abscessed formations in bone contact, and assessed the extent of the infection with swollen appearance of the masseter muscle and soft tissues on contact (Figure 1d–f). Intraoral drainage of the collection was performed under local anaesthesia and the patient went home after three days of inpatient care.

*Patient 2*

A 32-year-old woman came to the dental emergency department reporting swelling of the left cheek with insomnia-related pain for three days and difficulty eating. She had undergone surgery to remove two left wisdom teeth (teeth #28 and #38) six months earlier. Clinically, she presented a slight trimus, a swelling of the left buccal space and no fever. She had no sign of severity such as dysphagia or dyspnea. Her general practitioner had prescribed antibiotics (spiramycin/metronidazole combination) and a nonsteroidal anti-inflammatory drug (ibuprofen) two days prior. 

Pulp vitality tests were positive in the left posterior mandibular teeth and palpations at the left retromolar level were painful. A panoramic x-ray and a Cone Beam CT were performed to analyze the bone structures and to try to find the cause of the infection. Bone modification was observed in distal to the left mandibular second molar, possibly due to poor healing at the wisdom tooth extraction site. Finally, the most probable diagnostic hypothesis was an osteitis of the socket of the left mandibular wisdom tooth (#38) at the origin of the cellulitis. A complete blood count was performed with a slightly increased polymorphonuclear neutrophils and leucocytes (11,9 G/L and 14,9 G/L, respectively), and a slightly elevated C-reactive protein (7,2 G/L). The patient returned home with a prescription for antibiotics (amoxicillin and metronidazole); clinical and thermographic control at 48 h showed their effectiveness. A bone debridement procedure was performed a few days later and the anatomopathological analysis supported osteitis.

### 3.2. Thermal Analysis

*Patient 1*

**Figure 2:**
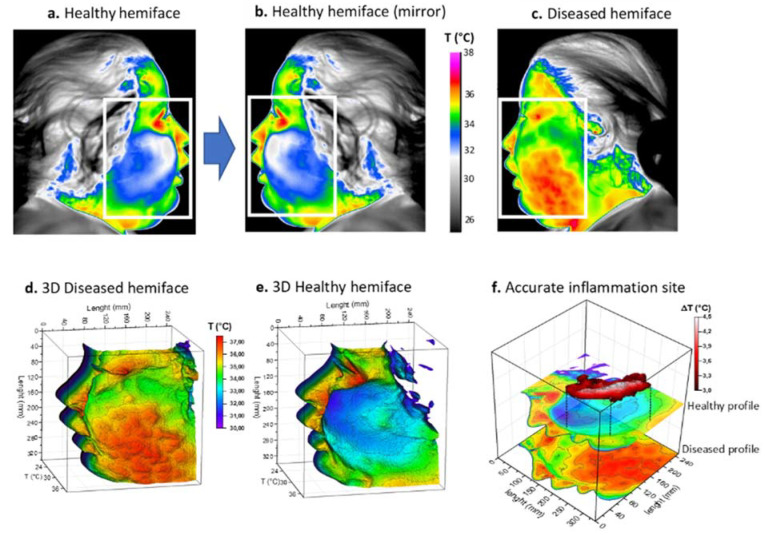
Thermal procedure of patient 1 with facial cellulitis. (**a**) Thermal image of the healthy hemiface with corresponding region of interest (ROI); (**b**) Returned (mirror) thermal image of the healthy hemiface; (**c**) Thermal image of the diseased hemiface with the same ROI; (**d**,**e**) 3D representations of the ROI weighted by temperature; (**f**) Image subtraction principle and resulting site of highest inflammation. White boxes correspond to the ROI.

Infrared thermal images were then acquired on the patient’s admission to hospital. The thermal maps in Figure 2 clearly show a large thermally activated area on the left side, compared to the contralateral healthy side of the face, with a difference of over 3 °C (Figure 2a–c). Because of the extent of the inflammation, a rectangular region of interest (ROI) was chosen with a matrix size of 350 × 250. A three-dimensional representation of the temperature fields is also provided in the same figure (Figure 2d,e). For this patient, the inflammation appeared to be more localized in the cheek, not reaching the orbital space. Inflammation in this patient was considered severe and the cut-off value of 3 °C was chosen for matrix subtraction (Figure 2f). The result showed an elongated, island-like shape of high temperature, corresponding to the main active site of inflammation.

**Figure 3:**
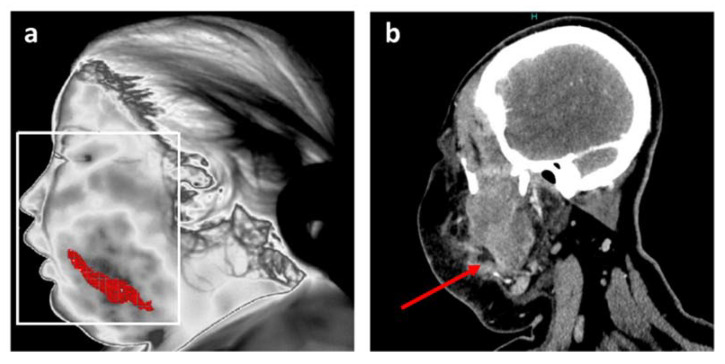
Position of the main inflammation site after thermal analysis for patient 1. (**a**) Resulting accurate localization of main inflammation linked to facial cellulitis with MIT; (**b**) Coronal plane of CT scan in soft tissue window at the level of the infectious collection (red arrow).

One of the original features of the adopted approach consists of its ability to reposition the inflamed areas detected on patients’ faces. The final result is shown in Figure 3a. Finally, thermal image processing confirmed that infection was limited to the buccal space without reaching any danger zone such as the eye or a more posterior zone such as around the pharyngeal axis. 

This information is in agreement with the data from the head and neck CT scan, and is notably comparable with a CT image in a coronal plane at the level of the infectious collection (Figure 3b). 

*Patient 2*

**Figure 4:**
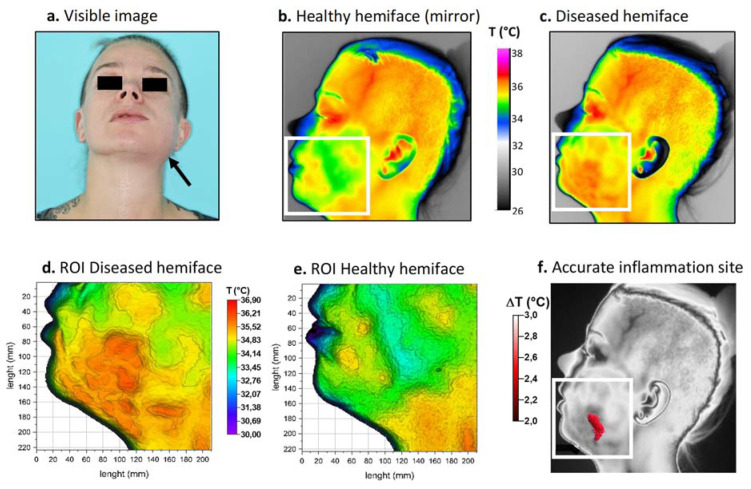
Thermal procedure in patient 2. (**a**) Front view visible image of the patient; (**b**) Returned (mirror) thermal image of the healthy hemiface with corresponding ROI; (**c**) Thermal image of the diseased hemiface with the same ROI; (**d**,**e**) 3D representations of the ROI weighted by temperature; (**f**) Resulting site of highest inflammation. White boxes correspond to the ROI.

Thermal images were again taken upon hospitalization. The colorimetric variations between the affected area (in orange-red) and the healthy area (in blue-green) corresponded to a temperature difference of approximately 2 °C (Figure 4b,c). For this patient, inflammation linked to cellulitis was thermographically visible on the lower left face and in the left submandibular region (Figure 4d,e). Because the inflammation level was considered moderate, a threshold value of 2 °C was chosen for matrix subtraction. The result (Figure 4f) showed a limited, comma-shaped area of high temperature located above the inferior border of the mandible corresponding to the main site of inflammation. Thermal image processing confirmed that infection was limited to the buccal space.

## 4. Discussion

Facial cellulitis is a common infection of head and neck fascial spaces mainly attributable to dentoalveolar origin, such as dental infections, postoperative infections, periodontal disease or inflammation of the pericoronal tissues [9,10]. Decisions on whether to hospitalize patients in order to treat acute odontogenic facial cellulitis (AOFC) usually rely on the severity of the infection [6,9]. Three aspects largely determine the severity and spread of infectious process: (i) the warning clinical signs (high fever > 38.5 °C, weakness, dyspnea, dysphagia, severe trismus, edema of eyelids, impaired vision, etc.), (ii) the number of infected anatomical spaces (Flynn score), and (iii) the causative tooth (upper or lower, anterior or posterior teeth) [7]. The spread of AOFC through the different fascial spaces is usually by clinical and radiological examination [5,8]. 

Buccal cellulitis is usually associated with a low severity score and favorable prognosis. This infection presents as a diffuse swelling of the cheek, located between the superficial skin layer and the deep buccinator muscle, and from the corner of the mouth to anterior edge of the masseter [12]. The posterior teeth (i.e. premolars and molars) in the maxilla or mandible are most often responsible. However, infections in this space can sometimes spread rapidly to the infra-orbital space and, less frequently, to the submandibular and submasseteric spaces [8,12]. The infraorbital space contains the main venous drainage of the face (the angular vein of the facial vein), and communicates freely posteriorly with the orbit and deeper anatomic spaces of the skull base [30]. Infra-orbital cellulitis may be associated with significant complications, including a septic thrombophlebitis from the angular vein, orbital abscess formation, cavernous sinus thrombosis and sepsis [8]. The prevalence of orbital cellulitis with odontogenic cause is 2–5% [12]. Careful examination and palpation of the medial canthus and nasal sidewall are important to detect thrombophlebitis and extension of the odontogenic infection into the orbit. However, the detection of widespread or embolic cellulitis using palpation alone is quite subjective [12]. Palpation may occasionally be difficult to perform, e.g., with pediatric or bariatric patients [3,8]. Furthermore, the lack of clinical warning signs in the early phase of infection may lead to misdiagnosis and delayed surgical intervention or hospital care, leading to serious and potentially life-threatening complications [7]. In our overweight patient, the physical examination alone did not allow us to precisely identify the number of anatomical spaces involved. 

Diagnostic technology continues to evolve, especially MIT [16]. These improvements may be able to better determine the location and extent of the inflammatory process than clinical examination alone. Indeed, MIT makes it possible to locate the inflammatory focus related to the infection using internal thermal conduction within the surrounding tissues during facial cellulitis, and to distinguish diffuse buccal cellulitis spread into periorbital tissue and buccal cellulitis alone with a reactive palpebral edema. 

Previous studies have found an increase of temperature between infected wounds and normal tissues (1.4 °C to 4 °C). Local temperature in skin surface differences over 3 °C are more likely suggestive of infection [21,31]. Thermography could be used as an adjunct for making accurate differential diagnoses between AOFC and abscess [32], and could therefore be an efficient, noninvasive medical tool to enhance clinical examinations, i.e., assessments of the extent of cellulitis (essential in severity assessments to decide upon the need of hospitalization). Facial examinations using MIT are easy to implement, and the findings are reliable and reproductible. 

The human face lends itself well to thermographic study because all the essential areas can be analyzed [33,34]. Some anatomical areas have a high thermal intensity (such as the medial palpebral commissure, labial commissure, temporal, supratrochlear and external acoustic meatus), while other areas are identifiable by their low thermal intensity (such as the inferior labial, lateral palpebral commissure and nasolabial) [29]. Previous studies have also reported that in healthy people, temperature distribution is symmetrical between the two sides of the human body, while in diseased individuals, this thermal distribution is abnormal due to changes in blood flow [35]. Our work has the advantage of comparing complete anatomical areas, taking into account all the pixels of the area rather than only a few representative points, as performed in other studies [21]. The postprocessing procedure is based on the acquisition of thermal maps of regions of interest (ROIs) of the face, symmetrically located relative to the sagittal plane at head and neck level [21]. The idea was to carry out a thermal mapping to precisely determine the location of the main inflammatory site by subtracting the thermal data from the healthy hemiface to the diseased hemiface. 

Periapical, panoramic or even three-dimensional x-rays such as cone-beam computed tomography (CBCT) are easily carried out in dental emergency departments, but they are only capable of identifying the origin of the infection, providing no information about the surrounding soft tissues [12]. Computed tomography (CT) scans are an essential tool to evaluate the anatomical location of infections and their spread to surrounding and/or deeper spaces; they can be performed in a radiology office or hospital [5,9,36,37,38]. Several studies have demonstrated that CT scans are more accurate than clinical examination alone to distinguish between a drainable collection and cellulitis, as well as to find the odontogenic source and the causative tooth [8,12]. The need for dentists or emergency practitioners to request a CT scan in an emergency context is not always evident; this may hinder the implementation of a prompt and appropriate treatment [39,40]. Therefore, nonionizing and rapid MIT examination could be used easily for patients with suspected facial cellulitis requiring a CT scan. This examination would be particularly suitable for pediatric patients with parents who are reluctant to expose their child to radiation, but also for pregnant women, people with disabilities or bedridden individuals. 

Moreover, since prompt treatment of severe odontogenic infections promotes shorter hospital stays with fewer complications [6,40,41], MIT could become a tool for clinical decision support for physicians regarding the need of cervico-thoracic CT scans. Biological evaluations using blood analyses of the C-reactive proteins and numbers of white blood cells, which are predictive factors of severe complications of facial cellulitis, could also be requested [5,10,42,43,44]. Lastly, thermography could be used for thermal monitoring in the evolution of cellulitis over time to observe, for example, the effectiveness of an antibiotic treatment. 

In the case of patient 1, based on visual and usual clinical considerations, the medical staff decided to hospitalize the patient. Independently, thermal scientists were asked to develop and analyze infrared thermal images of the inflamed diseased areas to see if new information could help in deciding whether to hospitalize or not. Retrospectively, the accurate localization by thermal analysis of the inflammatory site showed that hospitalization of this patient was not necessary. The complementary use of the MIT could thus provide additional information to guide choices made by medical staff during clinical examinations regarding the need for hospitalization. Following this preliminary case, it will be important to reproduce this methodology on other patients with different localizations of cellulitis, and to evaluate the sensitivity and specificity of the proposed technique compared to other current medical imaging systems such as CT scans and USG. The image processing could be systematized using software to interpret the data from the thermal camera in order to have a response in real time. This tool could be particularly suitable for rapid evaluations of progressing cellulitis, such as gangrenous cellulitis, where gas could be visualized [45], or in cases of facial necrotizing fasciitis [8]. 

Furthermore, MIT yielded clinically relevant information regarding the determination of an optimal approach of surgical drainage. Some authors have suggested that a prompt and accurate incision in the area of infection for drainage would be beneficial in the resolution of odontogenic infections [7,9]. The mapping of skin temperature in superficial fascial spaces provides additional valuable information which could guide percutaneous needle aspiration (for soft tissue decompression and microbiological testing), or incision, warranting drain positioning in the correct fascial space, with a possibly intraoperative check [38]. 

## 5. Limitations

The main limits of MIT use in the diagnosis and management of AOFC are its failures (i) to precisely identify the infected causative tooth, and (ii) to evaluate the spread of infection into the deep cervical and fascial spaces. Moreover, although AOFC are predominantly lateral and hemifacial, the image technical process described here does not allow median and paramedian dental cellulites to be treated in the same way. However, anatomical spaces which are at risk of complication are mainly located laterally [5]. Another limitation concerns thermal biases that could exist in cases of strong facial deformity, which can occur when medical care is implemented too late. In such cases, the juxtaposition of the two ROIs may appear too random due to the strong convexity of the diseased surface, and thermal errors may occur regarding the determination of the skin emissivity when the angle between the camera axis and the normal surface becomes too great. An additional limitation of our method is its ability to thermally explore only relatively superficial spaces. Any deep space infection, especially in the skull or retropharyngeal area, cannot be detected. However, inflammation at relatively deep tissue levels still diffuses heat to the surface [46].

## 6. Conclusions

To conclude, with promising early outcomes, medical infrared thermography could become a tool for clinical decision making for patients with acute odontogenic facial cellulitis by improving the performance of clinical extension assessments of the tissues of the head and the neck with the aim to optimize care. Indeed, this nonionizing medical imaging technique could help in decision-making regarding the need for CT scans or emergency hospitalization, as well as guiding incisions for drainage of infection. Thermal imaging cameras are now more accurate and affordable. Additionally, they are now easy to use, requiring only a few minutes to map and compare temperatures, making them a complementary tool to clinical examinations that could easily be adopted in emergency departments. 

## Figures and Tables

**Table 1:** Clinical information of patients.

Clinical Characteristics	Patient 1	Patient 2
Age (years-old)	25	32
Sex	Female	Female
Comorbidity	Overweight	-
Anatomical location of cellulitis	Left buccal space	Left buccal space
Fever	38.7 °C	No
Dolor	Moderate	Severe
Other symptoms	Severe trismus, difficulty to open the left eye	Mild Trismus
Medical treatment before thermal analysis	No	Antibiotics (spiramycin/metronidazole), Ibuprofen
Biological examination	-	Increase of polymorphonuclear neutophils and C-reactive protein
Causative factor	Left maxillary wisdom tooth (#28)	Left mandibular wisdom tooth (#38)
Additional radiological examination	CT scan of face and neck	Cone Beam CT
Hospitalization	Yes	No
Care	Intravenous antibiotics and drainage	Oral antibiotics (amoxicillin/metronidazole) and surgical debriefing
